# Fournier's Gangrene: Report of 2 Cases

**DOI:** 10.1155/2012/984195

**Published:** 2012-02-09

**Authors:** Prasan Kumar Hota

**Affiliations:** Department of Surgery, Institute of Medical Sciences & SUM Hospital, SOA University, K-8, Kalinga Nagar, Orissa Bhubaneswar 751030, India

## Abstract

Fournier's gangrene is a very serious surgical emergency seen all over the world. With the newer advancement of surgical techniques and critical care medicine, the mortality and morbidity of this disease has come down significantly over a period of time. An early diagnosis including evaluation of predisposing and etiological factors, metabolic and physiological parameters with prompt resuscitation, aggressive surgical debridement, broad-spectrum antibiotic coverage, and continuous monitoring of all the parameters is essential for a good outcome, therefore reducing the high mortality and morbidity of this condition. In this study, we report 2 different cases of Fournier gangrene. Our first case was a young, nondiabetic, and without any multiorgan failure, who was managed successfully with good outcome. The second case was a 67-year-old man with diabetes and multiorgan dysfunction with extensive gangrene at presentation, who recovered well, but with a stormy postoperative period.

## 1. Introduction

Fournier's gangrene (FG) is a serious surgical emergency. This clinical condition was first described by Jean Alfred Fournier (1832–1914), a dermatologist and venereologist. He had first described this condition in 5 young male patients, who had presented with a rapidly progressing fulminating infection of the superficial tissues of scrotum and penis without any definite etiological factor [1, 2]. It is interesting to note that Bauriene in 1764 had described such a case of scrotal gangrene due to traumatic injury from the horn of an ox, which was treated by multiple sittings of surgical debridement [3]. Over a period of time, the definition of FG was broadened to have all the necrotizing infections of the genitalia.

 At present, FG is recognized as a subclassification of necrotizing fasciitis. Hence, FG is described as necrotizing soft tissue infections originating from or limited to the genitalia or perineum irrespective of sex. We report 2 cases of FG, presenting in two different scenarios and their outcome.

## 2. Case Presentation

### 2.1. Case 1

**Figure 1:**
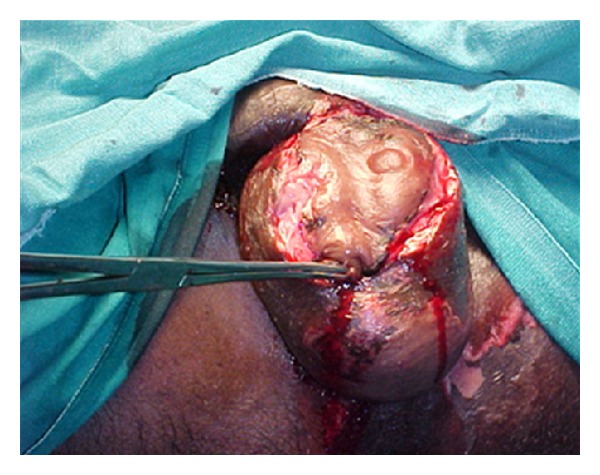
Fournier's gangrene.

**Figure 2:**
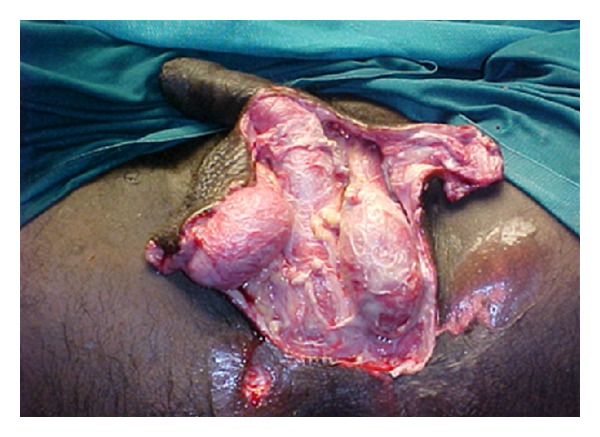
FG after debridement.

**Figure 3:**
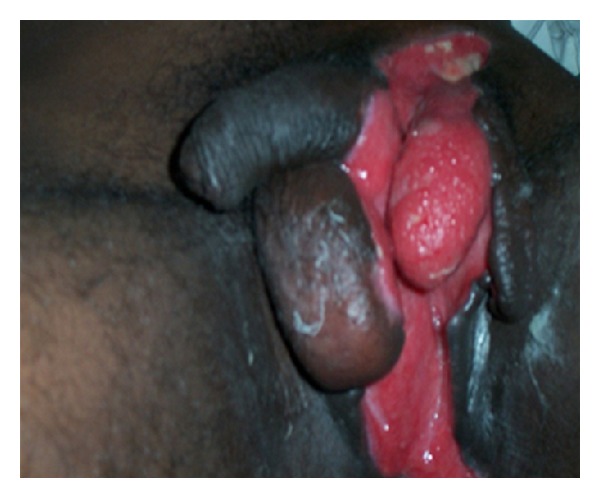
8th postop day.

**Figure 4:**
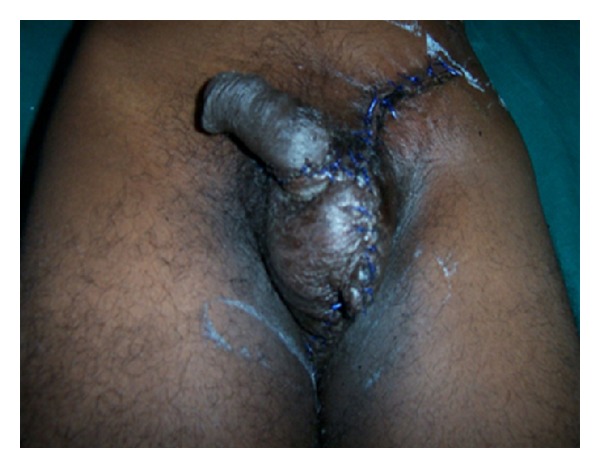
postreconstruction.

 27-year-old male patient reported to the hospital with complaints of painful swelling of the scrotum for 3 days and fever with discharge from the scrotum for 2 days. There was history of minor injury over left side of the scrotum 6 days back, for which he had not taken any treatment. There was no history of diabetes mellitus. Patient was not an alcoholic. He was not from a filarial endemic zone. On examination, he was conscious. There was no pallor, icterus, and lymphadenopathy. He was mild dehydrated. His pulse was 110/min, regular, and good volume. His blood pressure was 110/78 mm Hg. Systemic examination revealed no abnormality. Local examination of the scrotum revealed that scrotum was enlarged, edematous, and tender along with palpable crepitations. There was patchy gangrene all over the scrotum more over left side with foul-smelling purulent discharge (Figure 1). A provisional diagnosis of FG was made. The patient was resuscitated and investigated. Broad-spectrum antibiotics in form of cefoperazone and sulbactum along with metronidazole were started. He was prepared for emergency surgical debridement. Blood haemogram revealed hemoglobin (Hb%)—14 gm%, white cell count—19,500/cmm with polymorph nuclear leucocytosis (N-85%). Biochemical parameters were essentially normal (blood urea: 40 mg%, serum creatinine: 1.0 mg%, random blood sugar: 110 mg%, and LFT: within normal range). He was taken up for emergency debridement. All the devitalized tissue was excised (Figure 2). Pus was sent for culture and sensitivity test. Postoperatively patient was managed with broad-spectrum antibiotics and wet dressing. Culture revealed Staph.aureus and *E. coli* sensitive to cefoperazone and sulbactum. He responded to the treatment very well (Figure 3). Regular wet dressing was done along with topical application of povidone iodine. On 10th postoperative day, his wound was reconstructed with secondary suturing (Figure 4). He was discharged on 22nd postoperative day. A review after six weeks revealed the patient to be symptom free.

### 2.2. Case 2

Our second case was a 67-year-old male patient, a known diabetic on irregular treatment, reported with complaints of swelling, pain, and foul smelling discharge from the scrotum of 7 days duration. For this complaint, he was treated in a peripheral hospital, where his condition was deteriorated, for which he was brought to our hospital. On evaluation, he was found to have pallor, mild icterus, and moderate dehydration. His general condition was poor. His pulse was 130/min. Blood pressure was 100/50 mm Hg. He had tachypnoea. He was confused. Systemic examination revealed no clinical abnormality. Local examination revealed that his scrotum was grossly edematous with multiple discharging gangrenous patches all over. Scrotum was tender with palpable crepitations all over. The patient was vigorously resuscitated and investigated. Broad-spectrum antibiotics in form of cefoperazone and sulbactum along with clindamycin were started. An indwelling Foley's catheterization was done, and only 50 mL of concentrated urine was drained. History revealed that he had passed only 350 mL of high colored urine during previous 24 hours. Blood haemogram revealed hemoglobin (Hb%): 10 gm%, white cell count: 25,300/cmm with polymorph nuclear leucocytosis (N-90%). Biochemical parameters were deranged (blood urea: 65 mg%, serum creatinine: 2.5 mg%, random blood sugar: 450 mg%, LFT: serum billirubin: 2.8 mg%, AST: 75 IU/L, ALT: 65 IU/L, prothrombin time, and serum electrolyte—within normal range). ECG and X-ray chest revealed no abnormality. With the consultation of the endocrinologist, his high blood sugar was taken care of. He was taken up for emergency surgical debridement. Pus culture and sensitivity were asked for. Culture report showed *E. coli* and *Staph. aureus* sensitive to cefoperazone and sulbactum, cefotaxime and ciprofloxacin. Same antibiotic regime was continued. He had a stormy postoperative period. He developed pneumonia, for which he was adequately treated by the physician. His urine output became normal by 2nd post op day. By 5th post op day, his blood urea and serum creatinine came within normal range. His jaundice subsided, and by 7th postop day his liver function tests became normal. Blood sugar level was well controlled by insulin therapy. He required daily wet dressing and two more sittings of debridement. Healthy tissue was visible by 13th postop day. On 15th postop day, wound was reconstructed by secondary suturing. He was discharged from the hospital on 25th postop day.

## 3. Discussion

 FG is a serious surgical problem with high mortality and morbidity. Though there is a male predominance [4], this condition has been described in children also [5, 6]. Though we have not come across a single case in females, a recent publication shows a high incidence of 31.6% in female patients due to vulvar and Bartholin gland abscesses as well as in postoperative period following episiotomy and hysterectomy [7].

 There are so many predisposing factors described by various authors as seen in literatures. Out of them, diabetes, old age, alcoholism, obesity, paraplegia, and renal insufficiency are commonly seen. However, it is interesting to note that in almost 30% to 50% cases no definite predisposing factor is found [8]. The most commonly seen foci of infection are those arising from gastrointestinal tract (30% to 50%), genitourinary tract (20% to 40%), and cutaneous injuries and soft tissue (20%) [8]. In our experience mostly, we have come across cases of FG arising from minor injuries or soft tissue infection of scrotal skin.

 FG is commonly a polymicrobial infection of genitourinary or perianal source. However, the portal of entry is difficult to establish more often. Microbial invasion usually occurs either through direct injury or through a direct spread from urogenital organs or perforated viscus like colon, rectum, and anal orifice. In a meta-analysis, the portal of entry was found to be colorectal in 21%, dermatological in 19%, urogenital in 19%, where as in 36% of cases no definite portal of entry was established [4]. There are 3 types of necrotizing soft tissue infections seen in practice. Type I is polymicrobial in origin, where a combination of gram-positive and gram-negative bacteria along with anaerobes are seen in culture. Type-II infection is monomicrobial in nature, being usually caused by Group A streptococcus but may be associated with *Staphylococcus aureus*. Type II is less common as compared to Type I and usually seen in healthy, immunocompetent patients [9]. There is also a Type III infection caused by Vibrio vulnificus. We have never come across this variety.

 FG is a clinical diagnosis. In difficult situation, where a doubtful diagnosis exists, radiological evaluation becomes useful in its diagnosis. Plain radiography may show gas in the soft tissue [10]. Ultrasonography is very useful in detecting gas in the scrotal wall [11]. However, out of all the investigation modalities for diagnosis, CT scan has a greater value for evaluation of extent of the disease [12]. Out of all these investigations, ultrasound is a commonly available investigation modality, which can be done in every case to confirm the diagnosis and the extent of the disease process.

 Laboratory studies like white blood count presents a prognostic indicator at the time of presentation. Parameters, like low hematocrit, low-serum albumin, high blood urea nitrogen and serum creatinine, and high alkaline phosphatase have been shown as indicators for the mortality in various studies. Even hypercalcemia and increased serum lactate at the time of presentation have been found to be associated with mortality [13].

 Management of FG basically depends on multidisciplinary approach. Initial resuscitation with fluid therapy and restoration of cardiopulmonary function to normal in patients presenting with septic shock is very important at the time of presentation. Prompt and aggressive surgical debridement of devitalized tissue along with broad-spectrum antibiotics is the main stay of the treatment of FG. Antibiotics may be modified after obtaining the culture report. The removal of all the devitalized tissue is important to stop the progress of the infection and simultaneous elimination of systemic effects of toxins and bacteria [14]. Multiple sittings of surgical debridement may be required to achieve adequate local control of infection. Local wound care after surgical debridement is very important. Wet to dry dressings, dressings with vacuum-assisted closure devices (VAC dressing), and application of various topical agents have been advocated. We prefer daily wet dressing and topical application of povidone iodine. VAC dressings have shown enhanced granulation tissue and reduction in wound surface area compared to wet to dry dressing [15]. With proper surgical debridement, local wound care, and antibiotic therapy, healthy granulation tissue appears, and most of the time primary wound closure can be done, as seen in both of our cases. However in significant tissue loss, any of the reconstructive procedure including various flap covers may be considered depending on the case. A significant tissue loss in genitalia and perineum causing a large defect can lead to high morbidity, which can be salvaged by reconstructive surgery with adequate tissue coverage [16].

## 4. Conclusion

 FG is a serious surgical emergency with a high mortality rate. However, with the advancement in diagnostic modalities, surgical technique, potent antibiotics, and critical care, the morbidity and mortality of this dreaded clinical entity has decreased over a period of time. As a result of the improved approach of multimodality therapy, the mortality of FG in the hospital settings has decreased to 10 to 20%. We have presented here 2 cases of FG. Our first case was a young adult presented with FG without any associated comorbidity. He recovered well without any significant postoperative morbidity. Our second case was an old male patient, who presented with comorbidity like diabetes mellitus along with deranged multi organ functions. He had a stormy postoperative period with significant morbidity. He took a longer time to recover. In our experience, FG with diabetes mellitus always poses a greater challenge in reducing morbidity and mortality. It is recommended to adopt a multidisciplinary approach in treating a case of FG to achieve a low morbidity and mortality, especially in presence of the comorbidity like diabetes and multi organ failure.
